# The Incidence of Post-infectious Irritable Bowel Syndrome, Anxiety, and Depression in Iranian Patients with Coronavirus Disease 2019 Pandemic: A Cross-Sectional Study

**DOI:** 10.5152/tjg.2022.21651

**Published:** 2022-12-01

**Authors:** Farnaz Farsi, Sanaz Rezaei Zonooz, Zohreh Ebrahimi, Hanieh Jebraili, Mehrnaz Morvaridi, Tahereh Azimi, Masoumeh Khalighi Sikaroudi, Javad Heshmati, Soroush Khorrami, Marjan Mokhtare, Amirhossein Faghihi, Mohsen Masoodi

**Affiliations:** 1 Minimally Invasive Surgery Research Center, Iran University of Medical Sciences, Tehran, Iran; 2 Department of Nutrition, School of Public Health, Iran University of Medical Sciences, Tehran, Iran; 3 Department of Nutrition, Science and Research Branch, Islamic Azad University, Tehran, Iran; 4 Department of Health Sciences and Nutrition, Tehran University of Medical Sciences, Tehran, Iran; 5 Department of Nutritional Science, School of Nutritional Science and Food Technology, Kermanshah University of Medical Sciences, Kermanshah, Iran; 6 Colorectal Research Center, School of Medicine, Iran University of Medical Sciences, Tehran, Iran; 7 Colorectal Research Center, Iran University of Medical Sciences, Tehran, Iran

**Keywords:** Coronovirus disease 2019, hospital anxiety and depression scale, irritable bowel syndrome, Rome IV

## Abstract

**Background:**

Irritable bowel syndrome refers to a subgroup of disorders of gut–brain interaction associated with stress-related symptoms, but gastrointestinal infection can also be considered the leading risk factor. It is well reported that coronavirus disease 2019 can also result in gastroenteritis. Therefore, this study aimed to evaluate the incidence of post-infectious irritable bowel syndrome and stressful status among coronavirus disease 2019 patients.

**Methods:**

This cross-sectional study was conducted on adults with coronavirus disease 2019 referred to the Infectious Disease Clinic in Iran from November 2020 to February 2021. Patients who met all eligibility criteria were included in the study. The data were collected using a demographic questionnaire, Rome IV criteria questionnaire, and Hospital Anxiety and Depression Scale.

**Results:**

Totally, the data obtained from 233 eligible patients (136 women, 97 men; mean age 38.41) 11.52 (years) were collected and analyzed, and 53.2% of the cases had a moderate coronavirus disease 2019. The analysis showed that 27 (11.6%) patients suffered from irritable bowel syndrome symptoms based on Rome IV criteria after the recovery from the infection. Also, Hospital Anxiety and Depression Scale-based symptoms of depression and anxiety that occurred with coronavirus disease 2019 were reported in 27.4% and 36.9%, respectively.

**Conclusion:**

Our finding illustrated that irritable bowel syndrome symptoms based on Rome IV could occur in post-infected coronavirus disease 2019 patients. Also, Hospital Anxiety and Depression Scale-based symptoms of depression and anxiety were more common in females and coronavirus disease 2019 infected patients with clinical symptoms including cough, shortness of breath, and sore throat.

**Main Points**

- Irritable Bowel Syndrome (IBS) refers to a subgroup of disorders of gut–brain interaction, leading to a decline in the patient’s quality of life.
- Coronavirus disease 2019 (COVID-19), in addition to typical clinical manifestations (such as fever, myalgia or fatigue, myalgia or arthralgia, chills, low body temperature, and headache) could also result in gastrointestinal manifestations.
- Our findings demonstrated that post-infectious IBS and Hospital Anxiety and Depression Scale-based symptoms of depression and anxiety might occur in COVID-19 patients.

## Introduction

Irritable bowel syndrome (IBS) refers to a subgroup of disorders of gut–brain interaction characterized by various gut symptoms including, abdominal pain, diarrhea, constipation, changes in bowel movement, and cramps or bloating;^1,2^ its consequences negatively influence a patient’s quality of life and bring in increased global health care costs.^3^ Irritable bowel syndrome is the most commonly diagnosed gastrointestinal disorder worldwide. The global prevalence of IBS has been estimated to be between 4.1% and 5.5% according to the Rome IV criteria.^4-7^ A survey also reported that IBS prevalence among Iranian subjects aged 19-70 years had been about 21.5% (based on Rome III definition).^8^ The interaction between the gut–brain axis disturbances and genetic and psychosocial factors contribute to IBS development, but its pathogenesis, as a multifactorial disorder, remains unclear.^9,10^ Psychological stress acts as a trigger in developing IBS through its adverse effects on intestinal permeability and motility and hypersensitivity to visceral pain.^11^ What is more, physical and mental stress, in turn, could result in changes in gut microbiome composition, dysbiosis, and small intestinal bacterial overgrowth that could be involved in both IBS symptom origination and perpetuation.^1,12^ However, it is not yet clear whether the psychological characteristics are present before or after the onset of gastrointestinal symptoms.^13^

Today, the rapid severe acute respiratory syndrome coronavirus 2 (SARS-CoV-2) infection spread can be regarded as a primary global health concern all over the world.^14^ It has also been reported that coronavirus disease 2019 (COVID-19) caused millions of deaths in late 2019-2020 worldwide.^14^ This new acute respiratory syndrome, in addition to typical clinical manifestations, could also lead to gastrointestinal (GI) manifestations, including abdominal pains, diarrhea, nausea, and vomiting.^15^ A recent systematic review in this regard revealed that the prevalence of GI symptoms in COVID-19 patients is about 18% and is still increasing.^16^ Recently, reports suggest that the incidence of diarrhea varies from 2% to 20% in patients with COVID-19. Pan et al^17^ in 2020, examined the clinical characteristics of 204 patients with COVID-19 and observed the digestive symptoms, including diarrhea, vomiting, and abdominal pain in 50.5% of the cases.

Additionally, COVID-19 could adversely impress the nervous system of the GI tract, leading to hypomotility.^18^ It has been stated that COVID-19 can enter intestinal cells and cause gastrointestinal symptoms, despite the presence of low-pH gastric secretions and the secretion of bile and digestive enzymes in the GI tract.^19,20^ Detection of SARS-CoV-2 through oral and rectal swabs indicates that both gastrointestinal and respiratory tract can be targeted by SARS-CoV-2 together; which may represent a cross-talk between lungs and gut in COVID-19.^18,19^ It is also assumed that there is a relationship between the gut–lung axis and the severity of COVID-19 and dysbiosis.^20^ Meanwhile, the contribution of intestinal leakage to the severity of COVID-19 as a result of dysbiosis in the gut microbiome is not known.^18,20^

On the other hand, the COVID-19 pandemic could also have psychological effects, such as anxiety, frustration, uncontrolled fear, and disabling loneliness for people of all ages.^21^ As a result of these stressful situations, gastrointestinal dysfunction and other health complications following the coronavirus could be caused.^22^ Alzahrani et al^3^ demonstrated that IBS symptoms could exacerbate during the COVID-19 pandemic. It is well reported that COVID-19 infection can also result in gastroenteritis. Therefore, this cross-sectional study aimed to investigate IBS incidence and its correlation with stressful status among COVID-19 individuals after recovery of the disease.

## Materials and Methods

A prospective cross-sectional study was conducted on adults with COVID-19.^23^ They were referred to the Infectious Diseases Clinic of Hazrat Rasool Akram Hospital in Tehran (Iran) to diagnose SARS-CoV-2 infection based on clinical symptoms or due to the severity of the disease from November 2020 to February 2021. The participants undergoing a diagnostic procedure, such as a nasopharyngeal polymerase chain reaction (PCR) test (n = 136) or chest radiograph (n = 96) to show their lung involvement, had a definite diagnosis of coronavirus infection by their physician. The objectives of this study were explained in detail to all patients, and also, participating in this study was fully conscious and based on their desire. Further, written informed consent was obtained from each patient. Meanwhile, all patients received the national protocol of standard treatment without changes.

Patients with COVID-19 were investigated for the study inclusion or exclusion criteria after their recovery periods. The inclusion criteria were as follows: age 18 years old or above, having a definitive diagnosis of coronavirus with different severity (mild, moderate, and severe) using biochemical tests or computed tomography scan of chest and observation of lung involvement, absence of concomitant malignancies (such as cancer, pre-coronary kidney failure, heart failure, liver disorders, etc.). Exclusion criteria for this study were as follows: children, pregnant and lactating women, asymptomatic COVID-19 patients or individuals admitted to the ICU with critical conditions, having neurological diseases and gastrointestinal disorders, and an individual’s unwillingness to continue cooperation. Also, eligible participants with mild, moderate, and severe severity of coronavirus infection were included in the study. After at least 6 months of their recovery, all participants completed the stress and Rome IV questionnaires. The patients’ information, including (i) demographic data (such as age, gender, past medical history, drug history, and smoking); anthropometric measurements (such as height, weight, and body mass index (BMI)); (ii) biochemical test data (such as COVID-19-SARS-CoV-2 PCR, and anti-SARS-CoV-19 IgG and IgM); (iii) severity of illness based on physician diagnosis and symptoms (such as mild, moderate, and severe); (iv) clinical symptoms (such as fever, cough, shortness of breath, chest pain, redness of the conjunctiva of the eye, sore throat, loss of sense of smell or taste, shivering, pain, feeling exhausted, nausea and vomiting, and diarrhea) were also recorded at the baseline of the study. Notably, all obtained information of each patient was collected using telephone contact by our experts.

Rome IV questionnaire was applied to diagnose the types of IBS that assess recurrent abdominal pain or discomfort at least once a week for the past 6 months with at least 2 or more of the following features: (i) related to defecation; (ii) it is associated with changes in stool frequency; (iii) with a change in the shape (appearance) of stools.^23, 24^ In this study, the Hospital Anxiety and Depression Scales (HADs) questionnaire, a 14-item self-rating scale, was also used to evaluate anxiety and depression symptoms in different diseases related to psychological diseases. Each item has a score of 0-3; the higher rates show more significant anxiety or depression symptoms. The total scores in each section were classified from 0 to 21, which were as follows: the score from 0 to 7 showing a standard scale, 8 to 10 indicating a borderline range, and the scores between 11 and 21 representing clinical problems.^25,26^

The present study was also prepared following the ethical principles and confirmed by the medical ethics committee of Iran University of Medical Sciences, Tehran, Iran (No: IR.IUMS.REC.1399.514). All participants provided their written informed consent before the collection of the data. Data used for this study were extracted anonymously from the participants.

## Statistical analysis

The normality distribution of the variables prior to data analysis was examined by the graphical methods, numerical characteristics, and Shapiro–Wilk’s test. Descriptive statistics were also applied to express the qualitative variables using frequencies as numbers, and percentages and the value of continuous data were presented as mean and standard deviation. The independent sample *t*-test was carried out to compare the continuous variables. A chi-square test or Fisher’s exact test was also used to compare the categorical variables of characteristics in COVID-19 patients with and without IBS. Among the tertile of HADS in relation to depression and anxiety, we used a one-way analysis of variance for quantitative variables and a chi-square test for categorical variables. In the end, multivariable logistic regression was performed for COVID and IBS among tertiles of HADS scores. We calculated the odds ratio and 95% CI in adjusted models for age, sex, BMI, the severity of COVID, and past medical history. Statistical Package for the Social Sciences (SPSS) statistical program version 24 (IBM Corp., Armonk, NY, USA) was used to perform the statistical analysis. *P* < .05 was considered statistically significant.

## Results

**Figure 1.**
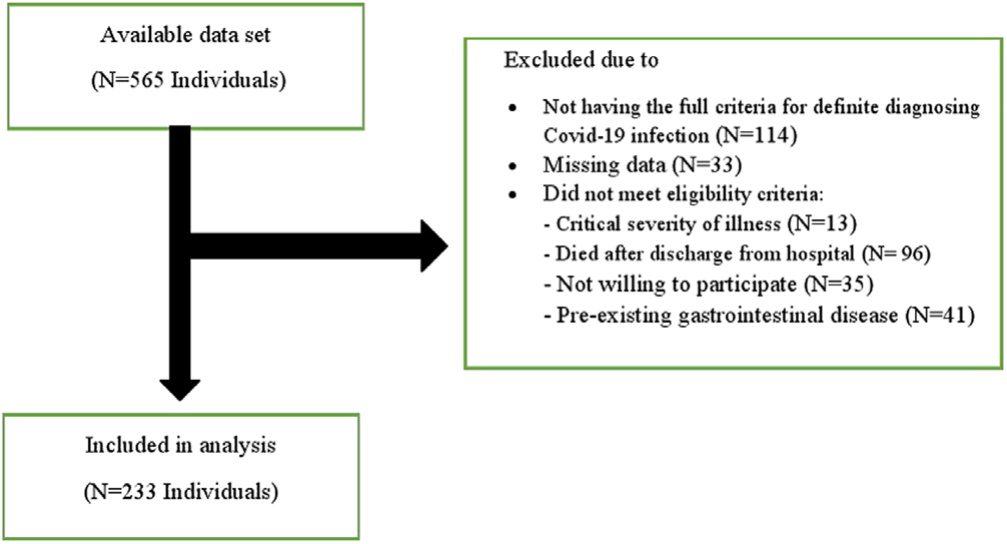
Flow diagram of study participants.

The current study assessed 565 patients with confirmed COVID-19 in terms of the study inclusion criteria. In total, 233 eligible patients were included in the study (Figure 1). The demographic characteristics of the participants are presented in Table 1. Of the 233 patients, 136 (58.4%) were females, and 97 (41.6%) were males. Also, the mean age and BMI of the participants were 38.41 (11.51) years and 26.29 (4.97), respectively. One hundred twenty-four (53.2%) patients had moderate COVID-19. As shown in Table 1, around 27 (11.6%) patients of all confirmed patients with COVID-19 experienced IBS symptoms based on Rome IV criteria. According to the result of HADS, symptoms of depression and anxiety in patients infected with COVID-19 were 27.4% and 36.9%, respectively. Most affected individuals had COVID-19 symptoms, such as fever, cough, body pain, and tiredness (Table 2). Table 3 shows the characteristics of the individuals reporting IBS symptoms after 6 months of being infected with COVID-19. The proportion of IBS occurrence was higher in women and people aged 40 years or over. In addition, there was no significant difference between the study participants in terms of severity of COVID-19, BMI, smoking, past medical history, and other factors. The characteristics of the participants among tertile of HADS in relation to depression and anxiety are reported in Tables 4 and 5, respectively. As illustrated in Tables 4 and 5, the trends of depression and anxiety in females were remarkably higher in tertile 3 versus tertile 1; meanwhile, notable declining trends of depression and anxiety were observed among males in the third tertile compared with the first-tertile. Accordingly, patients with fever, cough, shortness of breath, and sore throat experienced higher symptoms of depression. As shown in Table 5, a significant increasing trend of anxiety was shown among patients who had experienced cough, shortness of breath, and sore throat due to COVID-19. The logistic regression model results indicated that the odds of depression and anxiety were higher in COVID-19 patients with IBS symptoms, but the *P*-trend was not statistically significant (Table 6).

## Discussion

Based on the available literature, the COVID-19 pandemic had made significant lifestyle changes and stressful situations. This condition can lead to other acute and chronic diseases related to psychological stress and the nervous system like IBS.^27^ The main objective of this cross-sectional study was to evaluate the incidence of IBS and its correlation with stressful status in individuals who recovered from COVID-19. Our results demonstrated that the 27 COVID-19 patients (11.6%) suffered from post-infectious IBS symptoms according to Rome IV criteria; meanwhile, there was no significant relationship between anxiety or depression and the incidence of IBS symptoms. According to the obtained data, it seems that COVID-19 can directly or indirectly influence the gut function after the recovery by causing potential pathophysiological alterations including, dysbiosis, disruption of the intestinal barrier, intestinal inflammation, post-infectious states, gut–lung axis impairment, immune dysregulation, psychological stress, as well as use antibiotics, and other treatments of the acute phase.^28^ These are possible factors that can trigger IBS after the COVID-19 recovery, and infected individuals can generally be predisposed to the development of IBS. However, there is a paucity of data on the gastrointestinal sequelae of SARS-CoV-2 infection.^28^

Our results revealed that post-infectious IBS could develop in 11.6% of patients with COVID-19 based on Rome IV criteria. Wang et al^29^ also investigated the association of liver injury and gastrointestinal symptoms with the progression of COVID-19 in a systematic review and meta-analysis. Up to 53% patients had liver dysfunctions, 9.1% diarrhea, 5.2% nausea/vomiting, and 3.5% abdominal pain. The incidence of gastrointestinal symptoms in COVID-19 patients was relatively low, and it was not significantly associated with the COVID-19 progression.^29^ A retrospective study assessed the gastrointestinal manifestations of COVID-19 patients in Iran. The most prevalent gastrointestinal symptom was nausea/vomiting in 115 (18.8%) patients. In addition, there was not a relationship between respiratory symptoms and GI manifestations in the study.^30^ In line with these studies, Zhang et al^31^ investigated the clinical characteristics of COVID-19 patients with GI symptoms from Wuhan in early 2020. They reported that 164 patients (32.5%) experienced GI symptoms, including appetence, diarrhea, nausea, abdominal pain, and vomiting. In contrast, Noviello et al^32^ in 2021 assessed the IBS presence of 164 patients with SARS-CoV-2 infections versus a control cohort (n = 183) by applying the Rome IV questionnaire no increase in IBS among the post-COVID cohort compared to the controls. With these explanations, our observation should be interpreted with caution due to the low number of patients and the relationship between the GI symptoms in the acute phase of COVID-19 and post-infectious IBS. Therefore, further studies with higher statistical power would be needed to draw a firm conclusion.

Our reports also showed no significant impact of psychological stress on COVID-19-induced IBS symptoms in patients. In contrast, Oshima et al,^33^ in a population-based study involving 8.5% of patients with functional dyspepsia (FD), 16.6% with IBS, and 4.0% with FD-IBS overlap, revealed that the COVID-19 pandemic had adverse effects on both GI and psychological symptoms among individuals with FD-IBS overlap syndrome. Oshima’s study also indicated that the scores of anxiety and depression in the FD-IBS group were higher than those in the FD or IBS-only groups. Moreover, their results demonstrated that patients with FD-IBS overlap syndrome are more vulnerable to psychological stress due to the COVID-19 pandemic. Additionally, in a recent cross-sectional study, Kamp et al^34^ described the impact of the COVID-19 pandemic on psychological distress and gastrointestinal symptoms among 55 patients with IBS and comorbid anxiety or depression. The findings demonstrated that the COVID-19 pandemic increased psychological stress (67-92%) and GI symptoms (44-48%) among IBS patients. These differences in the results may be due to the small sample size and study location. On the other hand, a lack of information about patients’ psychological stress and GI symptoms before COVID-19 infection and during hospitalization could affect our findings.

The results obtained from this study also indicated that 36.9% of patients experienced stress and 27.4% suffered from depression, and patients with severe symptoms including fever, cough, shortness of breath, and sore throat experienced a higher rate of depression and anxiety. Zandifar et al^35^ reported that the prevalence of stress and depression in Iranian hospitalized patients with COVID-19 were 97.1% and 97.2%, respectively. The results of another study in London showed that 13.8% and 10.5% of COVID-19 patients discharged from the hospital were screened positive for depression and post-traumatic stress disorder.^36^ These studies’ differences in reported prevalence values revealed that hospitalization is a potential source of psychological disorders for COVID-19 patients.^37^ What is more, different tools for measuring psychological stress affected the reported prevalence values.

The other observations in our study suggested that depression and anxiety were more common in women than in men. Similarly, previous studies indicated that women experienced more stress in the COVID-19 pandemic, significantly influenced by poverty, housing insecurity, and other gender-based differences.^38^ Another study confirmed that being female, young, and single were associated with higher depression, anxiety, and stress.^39^ However, it could not be extrapolated to more severe effects of COVID-19 on the mental well-being of women. We did not have access to any baseline values known for anxiety and depression status for these patients. Even though psychological dysfunction after COVID-19 infection can be affected by various factors including level of exposure, loss of a family member, society, occupation, age, and gender.^40^

The strength point of this study was that the IBS incidence based on Rome IV criteria and its correlation with stress status in COVID-19 patients were investigated for the first time in Iran. However, there are some limitations, such as completing the questionnaire via telephone due to the pandemic situation, a small sample size, and lack of information about the stress status of subjects before COVID-19 infection.

## Conclusion

Our findings suggested that the post-infectious IBS based on Rome IV criteria might occur in 11.6% of COVID-19 patients and influence the recovery process of these patients. Furthermore, the HADS-based symptom of depression and anxiety were more common in women and COVID-19 infected patients with clinical symptoms, including cough, shortness of breath, and sore throat. Given the end of the COVID-19 pandemic, further studies with a larger sample size are needed to examine its short-term and long-term GI and psychological complications and find solutions for improving them.

## Figures and Tables

**Table 1.** Frequency of Sign and Symptoms in Patients During COVID-19 Infection

**Variables, n (%)**	**COVID-19 Patients (n = 233) **
**Fever**	
Yes	134 (57.5)
No	99 (42.5)
**Cough**	
Yes	125 (53.6)
No	108 (46.4)
**Shortness of breath**	
Yes	88 (37.8)
No	145 (62.2)
**Chest pain**	
Yes	82 (35.2)
No	151 (64.8)
**Redness of the conjunctive of the eye**	
Yes	37 (15.9)
No	196 (84.1)
**Sore throat**	
Yes	76 (32.6)
No	157 (67.4)
**Loss of sense of smell or taste**	
Yes	125 (53.6)
No	108 (46.4)
**Shivering**	
Yes	119 (51.1)
No	114 (48.9)
**Body pain**	
Yes	185 (79.4)
No	48 (20.6)
**Feeling exhausted**	
Yes	184 (79)
No	49 (21)
**Nausea and vomiting**	
Yes	67 (28.8)
No	166 (71.2)
**Diarrhea**	
Yes	79 (33.9)
No	154 (66.1)

Data are shown numbers (%).

COVID-19, coronavirus disease 2019.

**Table 2.** Demographic Characteristics of Post-infected Patients

**Variables**	**COVID-19 Patients (n = 233) **
**Age **(year) **^a^**	38.41 (11.51)
**Sex** ^b^	
Male	97 (41.6%)
Female	136 (58.4%)
**BMI ^a^**	26.29 (4.97)
**Weight** (kg)**^a^**	75.61 (18.27)
**Smoking** ^b^	
Yes	9 (3.9%)
No	224 (96.1%)
**Severity of COVID-19** ^b^	
Mild	91 (39.1%)
Moderate	124 (53.2%)
Severe	18 (7.7%)
**PMH** ^b^	
HTN	13 (5.4%)
DM	16 (6.6%)
Other disease	56 (24.5%)
**IBS** ^b^	
Yes	27 (11.6%)
NO	206 (88.4%)
**HADS-D** ^b^	
Negative	169 (72.5%)
Mild	35 (15%)
Moderate	18 (7.7%)
Severe	11 (4.7%)
**HADS-A** ^b^	
Negative	147 (63.1%)
Mild	41 (17.6%)
Moderate	21 (9%)
Severe	24 (10.3%)
**PCR test ^b^**	136 (58.4%)
Positive	
Chest radiograph ^b^	96 (41.4%)

^a^Data are shown as mean (SD)

^b^Data are shown in numbers (%).

BMI, body mass index; PMH, past medical history; COVID-19, coronavirus disease 2019; HTN, hypertension; DM, diabetes mellitus; HADS, Hospital Anxiety and Depression Scale; HADS-D, HADS-Depression; HADS-A, HADS-Anxiety; PCR, polymerase chain reaction; IgG, immunoglobulin G; IgM, immunoglobulin M; SD, standard deviation, SD; IBS, irritable bowel syndrome.

**Table 3.** Characteristics of Participants With and Without IBS

**Variables**	**COVID With IBS **(n = 27)	**COVID without IBS** (n = 206)	***P*** ^*^
**Age** (year)**^a^**	41.93 (14.34)	37.95 (11.05)	.09
**Sex** Male Female	10 (37%) 17 (63%)	87 (42.2%) 119 (57.8%)	.60
**Weight **(kg)**^a^**	73.55 (14.20)	75.88 (18.75)	.53
**BMI** (kg/m^2^)**^a^**	26.43 (3.99)	26.27 (5.09)	.87
**Severity of COVID-19 ^b^** Mild Moderate Severe	10 (37%) 13 (48.1%) 4 (14.8%)	81 (39.3%) 111 (53.9%) 14 (6.8%)	.33
**Smoking ^b^** Yes No	1 (3.7%) 26 (96.3%)	8 (3.9%) 198 (96.1%)	.96
**Past medical ^b^** Yes No	13 (48.1%) 14 (51.9%)	72 (35%) 134 (65%)	.18
**Fever ^b^** Yes No	18 (66.7%) 9 (33.3%)	116 (56.3%) 90 (43.7%)	.30
**Cough ^b^** Yes No	15 (55.6%) 12 (44.4%)	110 (53.4%) 96 (46.6%)	.83
**Shortness of breath ^b^** Yes No	14 (51.9%) 13 (48.1%)	74 (35.9%) 132 (64.1%)	.10
**Chest pain ^b^** Yes No	12 (44.4%) 15 (55.6%)	70 (34%) 136 (66%)	.28
**Redness of the conjunctive of the eye ^b^** Yes No	5 (18.5%) 22 (81.5%)	32 (15.5%) 174 (84.5%)	.69
**Sore throat ^b^** Yes No	7 (25.9%) 20(74.1%)	69 (33.5%) 137 (66.5%)	.43
**Loss of sense of smell or taste ^b^** Yes No	10 (37%) 17 (63%)	115 (55.8%) 91 (44.2%)	.06
**Shivering ^b^** Yes No	18 (66.7%) 9 (33.3%)	101 (49%) 105 (51%)	.08
**Body pain ^b^** Yes No	23 (85.2%) 4 (14.8%)	162 (78.6%) 44 (21.4%)	.42
**Feeling exhausted ^b^** Yes No	22 (81.5%) 5 (18.5%)	162 (78.6%) 44 (21.4%)	.73
**Nausea and vomiting ^b^** Yes No	7 (25.9%) 20 (74.1%)	60 (29.1%) 146 (70.9%)	.73
**Diarrhea ^b^** Yes No	13 (48.1%) 14 (51.9%)	66 (32%) 140 (68%)	.09

^a^Data are shown as mean (SD).

^b^Data are shown in numbers (%).

^*^Resulted from independent *t*-test for quantitative variables and chi-square for test for categorical variables.

BMI, body mass index; IBS, irritable bowel syndrome; COVID-19, coronavirus disease 2019.

**Table 4.** Characteristics of Participants Among Tertile of HADS in Relation with Depression

**Variables ^b^**	**HADS**-**D**			***P*** ^*^
	**Tertile 1** (n = 80)	**Tertile 2** (n = 89)	**Tertile 3** (n = 64)	
**Sex** Male Female	37 (46.3%) 43 (53.8%)	42 (47.2%) 47 (52.8%)	18 (28.1%) 46 (71.9%)	.03**^§^**
**IBS** Yes No	11 (13.8%) 69 (86.3%)	6 (6.7%) 83 (93.3%)	10 (15.6%) 54 (84.4%)	.18
**PMH** Yes No	25 (31.3%) 55 (68.8%)	34 (38.2%) 55 (61.8%)	26 (40.6%) 38 (59.4%)	.46
**Fever** Yes No	46 (57.5%) 34 (42.5%)	41 (46.1%) 48 (53.9%)	47 (73.4%) 17 (26.6%)	.003**^§^**
**Cough** Yes No	36 (45%) 44 (55%)	44 (49.4%) 45 (50.6%)	45 (70.3%) 19 (29.7%)	.006**^§^**
**Shortness of breath**				.01**^§^**
Yes No	27 (33.8%) 53 (66.3%)	27 (30.3%) 62 (69.7%)	34 (53.1%) 30 (46.9%)	
**Chest pain**				.001**^§^**
Yes No	24 (30%) 56 (70%)	22 (24.7%) 67 (75.3%)	36 (56.3%) 28 (43.8%)	
**Loss of sense of smell or taste** Yes No	45 (56.3%) 35 (43.8%)	46 (51.7%) 43 (48.3%)	34 (53.1%) 30 (46.9%)	.83
**Redness of the conjunctive of the eye** Yes No	11 (13.8%) 69 (86.3%)	14(15.7%) 75(84.3%)	12 (18.8%) 52 (81.3%)	.71
**Sore throat** Yes No	22 (27.5%) 58 (72.5%)	26 (29.2%) 63 (70.8%)	28 (43.8%) 36 (56.3%)	.08
**Shivering** Yes No	46 (57.5%) 34 (42.5%)	39 (43.8%) 50 (56.2%)	34 (53.1%) 30 (46.9%)	.19
**Body pain** Yes No	61 (76.3%) 19 (23.8%)	68 (76.4%) 21 (23.6%)	56 (87.5%) 8 (12.5%)	.17
**Feeling exhausted** Yes No	63 (78.8%) 17 (21.3%)	67 (75.3%) 22 (24.7%)	54 (84.4%) 10 (15.6%)	.39
**Smoking** Yes No	3 (3.8%) 77 (96.3%)	5 (5.6%) 84 (94.4%)	1 (1.6%) 63 (98.4%)	.43
**Nausea and vomiting** Yes No	17 (21.3%) 63(78.8%)	30 (33.7%) 59 (66.3%)	20 (31.3%) 44 (68.8%)	.17
**Diarrhea** Yes No	29 (36.3%) 51 (63.8%)	32 (36%) 57 (64%)	18 (28.1%) 46 (71.9%)	.51
**Severity of COVID-19** Mild Moderate Severe	29 (36.3%) 47 (58.8%) 4 (5%)	39 (43.8%) 43 (48.3%) 7 (7.9%)	23 (35.9%) 34 (53.1%) 7 (10.9%)	.50

Data are shown in numbers (%).

^*^ Resulted from ANOVA for quantitative variables and chi-square for test for categorical variables.

**^§^** *P*-trend <.05 was considered significant.

HADS, Hospital Anxiety and Depression Scale; HADS-D, HADS-Depression; BMI, body mass index; IBS, irritable bowel syndrome; PMH, past medical history; COVID-19, coronavirus disease 2019.

**Table 5.** Characteristics of Participants Among Tertile of HADS in Relation with Anxiety

**Variables**	**HADS **(Anxiety)			*P* ^*^
	**Tertile 1 **(n = 94)	**Tertile 2** (n = 72)	**Tertile 3** (n = 67)	
**Sex** Male Female	49 (52.1%) 45 (47.9%)	29 (40.3%) 43 (59.7%)	19 (28.4%) 48 (71.6%)	.01**^§^**
**IBS** Yes No	10 (10.6%) 84 (89.4%)	8 (11.1%) 64 (88.9%)	9 (13.4%) 58 (86.6%)	.85
**PMH** Yes No	31(33%) 63 (67%)	26 (36.1%) 46 (63.9%)	28 (41.8%) 39 (58.2%)	.51
**Fever** Yes No	49 (52.1%) 45 (47.9%)	42 (58.3%) 30 (41.7%)	43 (64.2%) 24 (35.8%)	.30
**Cough** Yes No	40 (42.6%) 54 (57.4%)	41(56.9%) 31 (43.1%)	44 (65.7%) 23 (34.3%)	.01**^§^**
**Shortness of breath**				.007**^§^**
Yes No	25 (26.6%) 69 (73.4%)	29 (40.3%) 43 (59.7%)	34 (50.7%) 33 (49.3%)	
**Chest pain**				.03**^§^**
Yes No	25 (26.6%) 69 (73.4%)	26 (36.1%) 46 (63.9%)	31 (46.3%) 36 (53.7%)	
**Loss of sense of smell or taste** Yes No	52 (55.3%) 42 (44.7%)	34 (47.2%) 38 (52.8%)	39 (58.2%) 28 (41.8%)	.39
**Redness of the conjunctive of the eye** Yes No	12 (12.8%) 82 (87.2%)	12 (16.7%) 60 (83.3%)	13 (19.4%) 54 (80.6%)	.51
**Sore throat** Yes No	27 (28.7%) 67 (71.3%)	17 (23.6%) 55 (76.4%)	32 (47.8%) 35 (52.2%)	.006**^§^**
**Shivering** Yes No	50 (53.2%) 44 (46.8%)	36 (50%) 36 (50%)	33 (49.3%) 34 (50.7%)	.86
**Body pain** Yes No	72 (76.6%) 22 (23.4%)	55 (76.4%) 17 (23.6%)	58 (86.6%) 9 (13.4%)	.22
**Feeling exhausted** Yes No	70 (74.5%) 24 (25.5%)	56 (77.8%) 16 (22.2%)	58 (86.6%) 9 (3.4%)	.17
**Smoking** Yes No	4 (4.3%) 90 (95.7%)	3 (4.2%) 69 (95.8%)	2 (3%) 65 (97%)	.90
**Nausea and vomiting** Yes No	23 (24.5%) 71 (75.5%)	23 (31.9%) 49 (68.1%)	21 (31.3%) 46 (68.7%)	.49
**Diarrhea** Yes No	32 (34%) 62 (66%)	31 (43.1%) 41 (56.9%)	16 (23.9%) 51 (76.1%)	.05**^§^**
**Severity of COVID-19** Mild Moderate Severe	41 (43.6%) 46 (48.9%) 7 (7.4%)	28 (38.9%) 39 (54.2%) 5 (6.9%)	22 (32.8%) 39 (58.2%) 6 (9%)	.73

Data are shown in numbers (%).

^*^Resulted from ANOVA for quantitative variables and chi-square for test for categorical variables.

**^§^** *P*-trend <.05 was considered significant.

HADS, Hospital Anxiety and Depression Scale; HADS-A, HADS-Anxiety; IBS, irritable bowel syndrome; PMH, past medical history; COVID-19, Coronavirus Disease 2019.

**Table 6.** Multivariate Adjusted Odds Ratio and 95% CI for COVID-19 Among Tertiles of HADS Scores (N = 233)

	**HADS-D**			***P*** **-trend** ^*^	**HADS-A**			***P*** **-trend** ^*^
	Tertile 1	Tertile 2	Tertile 3		Tertile 1	Tertile 2	Tertile 3	
OR (95% CI)	1	0.47 (0.16-1.36)	1.09 (0.41-2.84)	.55	1	1.07 (0.39-2.93)	1.22 (0.45-3.32)	.59

^*^Resulted from logistic regression. Adjusted for age, sex, BMI, the severity of COVID, and past medical history.

OR, odds ratio; HADS-D, HADS-Depression; HADS-A, HADS-Anxiety; IBS, irritable bowel syndrome; COVID-19, coronavirus disease 2019; HADS, Hospital Anxiety and Depression Scale.
